# Foetal Haemoglobin, Erythrocytes Containing Foetal Haemoglobin, and Hematological Features in Congolese Patients with Sickle Cell Anaemia

**DOI:** 10.1155/2012/105349

**Published:** 2012-07-05

**Authors:** L. Tshilolo, V. Summa, C. Gregorj, C. Kinsiama, J. A. Bazeboso, G. Avvisati, D. Labie

**Affiliations:** ^1^Unité de Dépistage de la Drépanocytose, Centre Hospitalier Monkole, BP 817, Kinshasa XI, Democratic Republic of Congo; ^2^Centre de Formation et d'Appui Sanitaire (CEFA), 10, Avenue Kemi, Mont Ngafula, Kinshasa, Democratic Republic of Congo; ^3^Servizio di Ematologia, Università Campus Bio-Medico di Roma, 21, Via Alvaro del Portillo, 00128 Roma, Italy; ^4^INSERM, Institut Cochin, 4, rue du Faubourg Saint-Jacques, 75014 Paris, France

## Abstract

High HbF levels and F cells are correlated with reduced morbidity and mortality in sickle cell disease (SCD). This paper was designed to determine the HbF and F cells levels in Congolese sickle cell anemia (SCA) patients in order to determine their impact on the expression of SCD. *Population and Method*. HbF levels were measured in 89 SCA patients (mean age 11.4 yrs) using a standard HPLC method. F cell quantitation was done in a second group of SCA patients (*n* = 42, mean age 8.9 yrs) and compared with a control group (*n* = 47, mean age 5 yrs). F cells were quantified by a cytofluorometric system (MoAb-HbF—FITC; cut off at 0.5%). *Results*. The mean value of HbF was 7.2% ± 5.0 with heterogeneous distribution, most patients (76%) having HbF < 8%. Mean values of F-cells in SCA patients and control group were 5.4% ± 7.6 (median: 2.19%; range 0,0–30,3%) and 0.5% ± 1.6 (median 0.0, range 0–5.18), respectively. SCA patients with F cells >4.5% developed less painful crisis and had higher percentage of reticulocytes. *Conclusion*. Congolese SCA patients displayed low levels of HbF and F-cells that contribute to the severity of SCD.

## 1. Introduction

Fetal hemoglobin (HbF, a2.2) is a major contributor to the phenotypic heterogeneity of sickle cell anemia (SCA). A major ameliorating factor is an inherent ability to produce fetal hemoglobin; elevated levels are correlated with reduced morbidity and mortality in patients with SCA [1–3].

In normal adults, HbF levels are distributed in a nonuniform way in the red cells with a range varying from 0.1 to 7% of total hemoglobin (Hb). In red cells producing higher HbF (termed F cells), HbF is elevated (around 25% of cellular Hb) and genetically determined [2, 4, 5].

Genetic variation at three principal loci—the HBB cluster on chromosome 11p, HBS1L-MYB region on chromosome 6q and BCL11A on chromosome 2p—have been shown to influence HbF levels and disease severity in *β* thalassemia and SCA. Taking into account these loci, there is still substantial residual variance in HbF levels, suggesting the importance of other quantitative trait loci (QTL) modulating HBG expression [4, 6].

Total Hb and HbF levels vary in SCA patients according to the *β*S haplotypes: values are greater in patients bearing the Arabo-Indian and Senegalese haplotypes and less in those with the Bantu or Central African haplotype [2, 4, 6–8].

Sickle cell disease (SCD) has a high prevalence in Sub-Saharan Africa where majority of the affected patients live. B^S^ gene prevalence in the Democratic Republic of Congo (DRC) is around 25% and about 1.7% of newborns are affected (50,000 births per year) [9].

Expression of SCA in Congolese patients displayed a severe form with high mortality and complications [10].

To our knowledge, there are no specific data on HbF and F cells reported in SCA patients living in DRC. We therefore present here the preliminary analysis of HbF and F cells in two series of SCA patients and correlations with other hematological parameters and clinical data.

## 2. Population and Methods

All patients were SS homozygotes regularly followed up in comprehensive sickle cell programs in DRC.

A first study quantified HbF in steady state SCA patients followed up in Lubumbashi (*n* = 48) and Kinshasa (*n* = 41), a total of 89 patients (34 M, 48 F; mean age 11.4 yrs ± 5.4). No patient was on hydroxyurea treatment.

The second study involved 42 SCA patients (23F, 24 M; mean age 8.8 yrs ± 5.1) and a control group of 47 non-SCA patients (26 F, 21 M; mean age 5 yrs ± 5.1) recruited in Kinshasa. In this study, we assessed F Cells numbers and compared the results to hematologic parameters and clinical data.

Diagnosis of SCA was established using standard hemoglobin electrophoresis techniques (acetate electrophoresis or Isoelectric focusing-IEF) coupled to Itano solubility test. The percentage of HbF was determined by high performance liquid chromatography (HPLC).

HbF expression was evaluated using a previously described flow cytometric procedure [11, 12] with slight modifications. In brief, twenty microliters of whole blood were fixed with 1 mL ice-cold 0.05% glutaraldehyde in PBS pH 7.4 vortexed for 15 seconds (s), then incubated at room temperature (RT) for 10 min. The cells were washed twice with PBS, permeabilized by vortexing for 15 s with 0.5 mL ice-cold 0.1% Triton X-100 (Sigma, Milan) in 0.1% bovine serum albumin in PBS (BSA-PBS), and incubated at RT for 5 min. The cells were then washed once with 0.1% BSA-PBS and suspended in 0.5 mL 0.1% BSA-PBS.

Ten microliters of cell suspension were then mixed with 20 *μ*L of 1-in-5 diluted MoAb-HbF-FITC (IQ products, Milan) in 0.1% BSA-PBS and 70 *μ*L of 0.1% BSA-PBS and incubated in the dark at RT for 15 min. An irrelevant mouse antibody of the appropriate subclass was used as a negative control to determine background fluorescence. The cells were washed once with 0.1% BSA-PBS and immediately measured by flow cytometry (as described below).

The flow cytometer analysis reported the percentage of F+ cells in total counted red blood cells of each sample. The positive cut off point was set at 0.5% above negative population of isotype control staining cells.

HbF expression was, also, analyzed using the Kolmogorov-Smirnov statistic test (*D*-value), which allows the objective and accurate identification of small differences in fluorescence intensity [13]. Samples with *D* < 0.15 were considered negative, whereas those with a *D* ≥ 0.15 were considered positive.

Modified technique for evaluating HbF expression: considering the complexity of the previous procedure for identifying the F+ cells, we applied a second flow cytometric technique to perform F+ cells evaluation. This method (routinely utilized for characterizing other cellular parameters, as for example MDR in patients affected by acute leukemias) enabled us to test the samples more conveniently, using fewer and simpler steps, and a precise identification of the red blood cells population in the flow cytometric dot plot, useful for a specific analysis. In addition, this technique led to increased capacity to analyze more samples together than the previous one.

Twenty microliters of whole blood were fixed (Fix and Perm permeabilization kit; Caltag Laboratories) with 100 *μ*L of Medium A at room temperature (RT), in the dark, for 15 min; then cells were washed once with PBS, and then after incubated with 100 *μ*L of Medium B and 4 *μ*L of MoAb-HbF-FITC at RT, in the dark for 30 min. Finally, cells were washed once with PBS, and immediately measured by flow cytometry. The flow cytometric analysis was performed considering the same parameters used for the previously described technique [11, 12].

*Flow cytometric analysis* was conducted using a FACScan flow cytometer (Becton Dickinson), operated at 488 nm which detects green (MoAb-HbF-FITC) fluorescence. Data acquisition and analysis were performed with the CellQuest software (Becton Dickinson). We measured 50,000 events. The red blood cell area was gated by forward scatter signals (FSC) versus side scatter signals (SSC). The latter was measured using the logarithmic scales (log SSC).

Comparison of hematological parameters (Blood cell counts and HbF levels) were made with other reports of African SCA patients [14–17].

These studies were approved by the Local Ethnic Committees of the participating institutions, Campus Biomedico di Roma, and The CEFA/Centre Hopsitalier Monkole, in accordance with the Declaration of Helsinki.

*Statistical analyses* were conducted with a software program SPSS system (Version 12, Chicago). Results were expressed as the mean value and median value: standard deviation(SD). Comparisons of means were analysed by Students *t*-test, correlations by Pearsons test, and comparison between categorical variables by Chi square test or Fishers exact test (where appropriated).

HbF expression (*D*-value) and F+ cells were represented as dichotomized variable (positive versus negative). Data were analyzed using the two-sided Student's *t*-test to correlate results obtained by mean of the two different parameters of analysis and the two flow cytometric techniques, while Mann-Whitney *U*-test was used to measure the differences observed between positive and control groups.

Values were considered statistically significant when *P* < 0.05.

## 3. Results

### 3.1. Patients Population

In the first study of 89 SCA patients, the mean HbF% was 7.2% ± 5.0 (median 5.9; range 1–27.5%). It was 7% and 7.4% in the Lubumbashi and Kinshasa group, respectively (*P* > 0.05). Values of HbF were higher in females (7.4%, mean age 10.4 yrs) than in males (6.9%, mean age 9.2 yrs), but the difference was not statistically significant (Mann Whitney test chi square = 0.018, edree of freedom = 1, *P* = NS). Higher values were observed in children aged less than 3 yrs but no statistical differences were observed between the different age groups.

**Figure 1:**
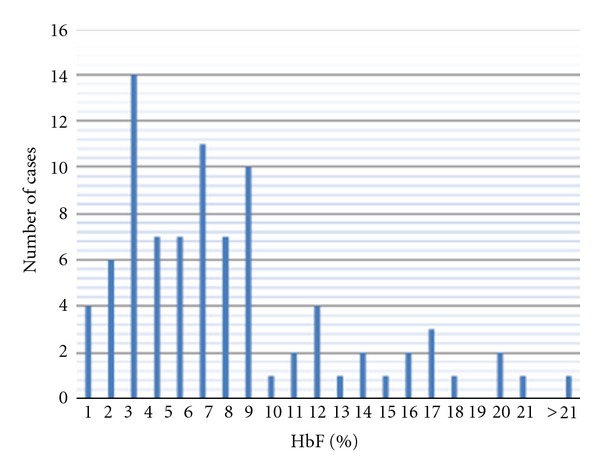
Population distribution of the HbF rate. Patient distribution related to HbF rate displayed a heterogeneous pattern with a predominant group (74%) with HbF% <8. GIobally, only 20/89 (or 22.5%) of patients displayed values higher than 10% of HbF.

Distribution of HbF rate displayed a heterogeneous pattern with a predominant group (66/89 or 74%) with HbF <8% and two other groups with 9–13% and 14–17%. Globally, levels of HbF were less than 10% in 69/89 of cases (77.5%) and varied considerably; the distribution pattern was not normal even after log transformation of values (not shown), (Figure 1).

**Figure 2:**
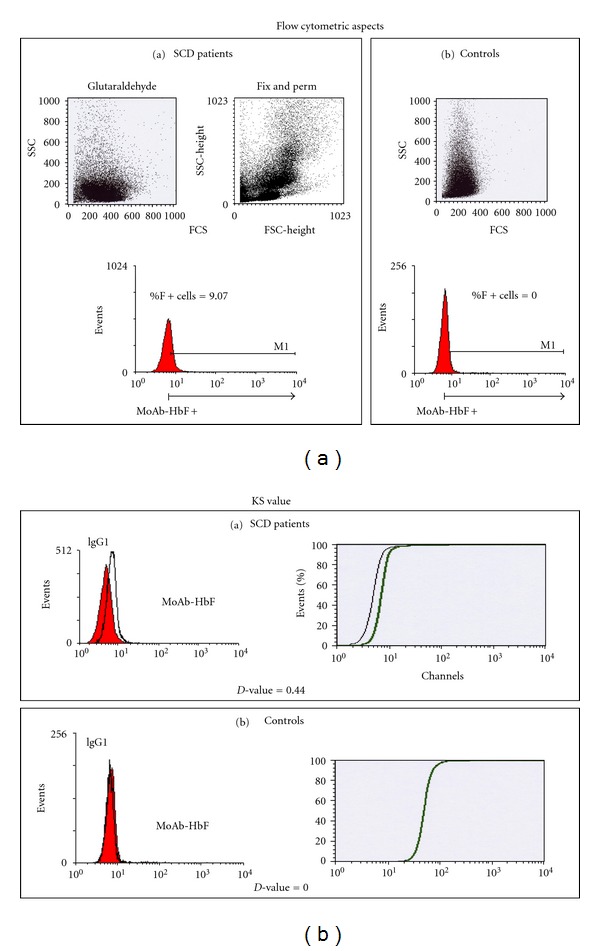
Illustration of flow cytometric and KS aspects of F cells values in a SCA patient and a control. HbF expression was, also, analyzed using the Kolmogorov-Smirnov (KS) statistic test (*D*-value), which allows the objective and accurate identification of small differences in fluorescence intensity. Samples were considered positive when *D* ≥ 0.15.

In the second study, enumeration of erythrocytes containing HbF (F cells) using the first, standard flow cytometric technique resulted in mean %. SD of F+ cells in 42 SCD samples of 5.44% ± 7.6 (median: 2.19%; range 0.00–30.3%). The mean *D*-value. SD was 0.21 ± 0.007 (median: 0.33; range 0.07–0.57). In the 47 controls, the mean % ± SD of F+ cells was 0.50% ± 1.06 (median: 0; range 0–5.18%), and the mean *D*-value ± SD was 0.024 ± 0.034 (median: 0; range 0–0.15) (Figure 2).

Correlation among F+ cells % and *D*-value for the entire population was highly significant with a *r* = 0.67 (*P* < 0.0001). The comparison among SCD patients and controls as for the % of F+ Cell and *D*-value was also highly significant (*P* < 0.001 for both F+ cells % and *D*-value).

The evaluation of the samples with the Fix & Perm flow cytometric technique showed a mean %. SD of F+ cells of 8.67%. 13.48 (median: 4.63; range 0–57.75%), while the mean *D*-value. SD was 0.19. 0.17 (median: 0.15; range 0–0.7).

Comparison of the two flow cytometric techniques showed strong correlation for F+ cells values (*r* = 0.63; *P* = 0.0005) and for *D*-value parameters (*r* = 0.53; *P* < 0.005). 40/42 of SCA patients (95%) had values above the cut-off value of 0.5% while in the control group, only 12/47 subjects (25.5%) had values above the cut off.

**Figure 3:**
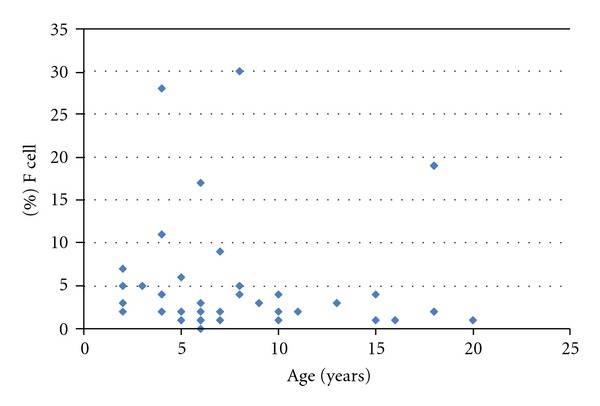
% F cells distribution in 42 SCA patients related to age. The mean value was at 5.44 ± 7.6 with a non gaussian pattern even after log-transformation of values (not shown).

Population distribution of % F cells were heterogeneous and displayed a nonnormal distribution even after log-transformation of values (Figure 3). Patients aged <12 yrs displayed higher values than older patients: mean values of 3.7 were observed in group 1 and 4.7 in group 2 versus 1.9 in group 3 (Table 1).

### 3.2. Comparison of %F Cells with Hematological Parameters and Clinical Issue

We found no significant correlations between the results obtained by cytometry system with the glutaraldehyde method with clinical and biological data; but with the Fix & Perm method, the number of vaso-occlusive crisis was significantly reduced in patients with F cells rate >4.5% (*P* < 0.05) while the reticulocytes number was significantly elevated (*P* < 0.005).

We did not observe significant differences between haematological parameters (Hb, MCV, MCH, and MCHC) in different age groups, although children aged >18 yr displayed higher value of RDW (Table 1).

Comparisons of hematological parameters in our patients with those described in other African SCA patients are depicted in Table 2.

## 4. Discussion

Hematological characteristics and clinical severity in SCA are variable and are influenced by environmental and genetic factors, including the presence of *α*-thalassemia, variation in Hb F level, and the haplotype background that is linked to the *β* globin gene [14]. The Bantu or CAR haplotype is considered as a major risk factor associated with clinically severe form of SCD and organ damage [7, 18, 19]. Most of the SCA patients living in central Africa and in DRC carry the CAR haplotype [20].

The protective role of HbF in the sickling of red cells and the clinical severity of SCD is evident. The HbF level has emerged as an important prognostic factor both for sickle cell pain and mortality; and a %HbF > 10% has been suggested as a threshold level for reduced clinical severity [5, 6, 19, 21, 22].

Different studies on HbF levels in SCA patients bearing CAR haplotypes reported levels values varying from 2 to 10.8%, but generally less than 10% [14, 18, 19, 23, 24]. To date, no values of HbF levels have been reported in Congolese SCA patients living in DRC. The mean value of 7.2% HbF observed in our study confirmed that patients bearing the CAR haplotype had levels of HbF less than 10%, the minimal level that permits a protective role on the sickling of red cells [2, 19]. We found no significant differences in Hb levels in our patients related to sex or age although recent studies confirmed that adult females have higher HbF and F Cells values than males because of the presence of an X-linked QTL (Quantitative Trait locus) [4, 6]. Mouele [16] reported similar data in the neigboring Congo Brazzaville.

Nagel et al. [19] suggested that the HbF level in SCA patients aged more than 5 yrs was dependent of the C-T mutation at position −158 G*γ* in the promoter of the G*γ* globin gene (known as the Xmn I-G*γ* site) [23]. They also found mean levels of HbF at 6.4% and 12.4% in the groups with a rate of G*γ* < 38% and G*γ* > 38%, respectively. The presence or absence of alpha deletion did not modify these observations. Patients with CAR haplotype had a low G*γ* globin gene expression in comparison to the other African haplotypes (Senegalese and Benin types) [19]. This polymorphism has been associated with erythropoietic stress and expanded erythroid mass secondary to ineffective erythropoiesis or hemolytic process and preferential survival of the red cell precursor that contain HbF, as observed in *β*-thalassemia and sickle cell anemia [4, 6, 24].

Genetic variation at three principal loci—HBB cluster on chromosome 11p, HBS1L-MYB region on chromosome 6q, and BCL11A on chromosome 2p—have been shown to influence HbF levels and disease severity in *β* thalassemia and SCA [4]. A recent study revealed that all three principal HbF loci have a significant impact in Tanzanian patients with SCA; the strongest association being seen at the BCL11A locus on chromosome 2 [25].

We think that values observed in our study were probably due to the heterogeneity of G*γ* globin gene expression in patient bearing the Bantu haplotype [19]. Comparison of the HbF values reported in other African SCA patients, displayed low values of 2,17 ± 1.81%, and 4.7 ± 2.9% in Nigerian patients described by Omoti [15] and Falusi and Olatunji [26], respectively. Mouele [16] reported values of 8.8 ± 5.8% in SCA patients in the neighboring Republic of Congo.

Differences observed in those African SCA patients may be due to the various age proportion of populations, the coinheritance trait of thalassemia gene, or other genetic components controlling the number of F cells and the clinical status of the patients [21, 24]. As HbF has been shown to be stable in SCA patients at 4–6 years [6, 19], comparison of HbF values should be determined in children aged >4 yrs and in steady state.

Levels of HbF and F cells vary considerably among different populations; this variability does not originate from a single genetic locus and HbF persistence is considered as a quantitative trait (QT) depending on multiple genes being expressed together with a small environmental component [4].

In normal adults, F cells % varied from 0.5 to 7% while in SCA patients, values have a much broader range [21]. We have no local reference values in Congolese SCA patients, but 40/42 (95%) of patients and 12/47 (25.5%) of controls displayed values higher than the cut-off point (0.5%). Higher values displayed by some SCA patients and controls (Figure 2) would be due to the concomitant hereditary persistence of fetal hemoglobin (HPFH) as described in other studies [21]. The wide ranging distribution of % HbF and F cells observed in our population can be due to the small sample size in this study, and also due to genetic factors (*α*-thalassemia and the QTL traits) and to environmental factors (malaria, infections). This later condition coupled with a chronic inflammatory status that has been reported in majority of SCA patients living in DRC [27] would contribute to the hyperhemolytic status. Hemolysis can play a role in the “erythropoietic stress” due to malaria and other infectious complications as expressed by the high reticulocytes number in Congolese SCA patients [28]. Further studies are required to evaluate this hypothesis.

Elsewhere, a significant correlation has been reported between the F cells rate and the %HbF and also with some erythrocyte indices like MCV and Hb [5, 21]. In our study, we found no significant correlations between F cells and other hematological parameters, except the reticulocyte percentage.

Although comparison of hematological parameters in SCA patients from different African countries showed variability, globally, it appears that SCA African patients had low values of Hb (<8 g/L) and %HbF (<10). These data can explain the severity of phenotype of SCD in patients bearing African *β* ^s^ haplotypes.

In spite of the wide individual variations of the %HbF and F cells rate, these two parameters would be used as a tool to monitor response to agents such as hydroxyurea, a drug that reduces the severity of SCD [4, 6, 21].

## 5. Conclusion

In spite of some limitations of this study, we have provided new data highlighting low HbF levels and clinical severity of SCA in Congolese patients which can be used to compare African patients located in different geographical area and genetic background. Moreover, comparison of both flow cytometric techniques for F+ cell quantitation resulted in a significant statistical correlations. To confirm its reliability, the less complex and quicker Fix & Perm technique should be further utilized for measuring the amount of F+ cells in SCA. Furthermore, Genome wide studies in different sub-Saharan SCA patients would contribute to the understanding of the complex role of HbF and F cells in the phenotype and the complex physiopathology of sickle cell disease.

## Figures and Tables

**Table 1:** Hematological parameters related to F cells rate in different age groups.

Group (n)	age (yrs)	WBC (G/L)	RBC (T/L)	Hb (g/dL)	Pcv (%)	MCV (fl)	MCH (pg)	MCHC (g/dL)	Pts (G/L)	RDW-cv (%)	PDW (%)	MPV	F Cell (D-value)	F cell %
1 (12)	2–5 (12)	18.38	2.49	6.4	21.8	89,9	26.3	29.2	514.8	24.5	13.1	10.1	0.3	3,7
2 (21)	6–12 (21)	18.74	2.39	6.4	21.1	90,6	27.3	30.2	434.5	24.2	12.6	10.1	0.3	4,7
3 (7)	13–18 (7)	15.92	2.12	5.8	18.6	87,9	27.1	31.0	367.0	25.9	14.5	11.4	0.2	1,9
4 (1)	>18 (1)	14.80	1.68	4.5	16.7	99,4	26.8	26.9	375.0	32.0	14.1	10.5	—	—

Subjects were divided in 4 age groups (1, 2, 3, and 4) and compared each to others. Parameters that displayed significant differences concerned the F cells rate and the RDW. Significant differences were observed in the F cells rate between the group 1 and 2 versus group 3 (*P* < 0.05); RDW was significantly higher in a child aged >18 yrs than in the other groups.

**Table 2:** Comparison of hematological parameters in SCA patients from different African studies.

Countries	Nb	Mean age	Hb (g/dL)	PCV (%)	RBC (T/L)	MCV (fl)	MCH (pg)	MCHC (g/dL)	HbF (%)	Ref erences
Tanzania	12	10.7	6.42	24.7	2.27	108.8	28.8	26	8.6	[14]
Kenya	25	10.9	7.85	26	2.54	102.4	30.9	30.2	7.5	[14]
Angola	4	9.3	7.30	20.7	2.70	88.3	30.5	35.3	2	[14]
Nigeria	249	9.7	7.53	28	2.76	103	26.8	26.8	9.2	[14]
Nigeria	94		7.4	26	3.6				7.2	[17]
Nigeria	200	23.6	7.5	23.0	—	79.3	28.3	32.5	2.1	[15]
R Congo	116	9.4	6.6						8.8	[16]
DR Congo	115	8.7	7.0	23.2	2.47	95.3	28.3	30.3	7.4	Personal communication
DR Congo	42	8.9	6.2	20.7	2.3	89.6	26.8	29.7	7.2	Our data

Most of the African SCA patients have Hb less than 8 g/L and Hb F less than 10%. Large variations of HbF rate were observed in the same country like Nigeria probably because of the heterogeneous population who were tested. In DR Congo, values were almost s similar.
